# Anti-CCP: History and its Usefulness

**DOI:** 10.1080/14767050500134217

**Published:** 2005-06

**Authors:** M. Herold, V. Boeser, E. Russe, W. Klotz

**Affiliations:** Rheumatology Unit Clinical Department of General Internal Medicine Innsbruck Medical University Innsbruck A-6020 Austria

## Abstract

Antibodies directed to cyclic citrullinated peptides (anti-CCP) are highly specific for rheumatoid arthritis (RA) and can easily be detected in sera by using commercially available immunoassays. The second version of the anti-CCP test (anti-CCP2) demonstrated high specificity (89-98%) and good sensitivity (41-88%) for RA. Commercially available ELISA methods from three different companies are on the market. All three CCP2 assays show similar results as all CCP2 assays use the same antigen-coated plates. This study was an evaluation of a new automated method for the determination of anti-CCP2 in a routine laboratory setting. Five hundred and fourty three serum samples were tested for anti-CCP2 within normal routine diagnostic using a commercially available ELISA and retested with a prelaunch version of a new and fully-automated method (*EliA*™). The results were comparable. The new automated assay is easy to use and demonstrated a diagnostic sensitivity of 80% and specificity of 97%.


*Clinical & Developmental Immunology*, June 2005; 12(2): 131–135

### Anti-CCP: History and its usefulness

M. HEROLD, V. BOESER, E. RUSSE, & W. KLOTZ

*Rheumatology Unit, Clinical Department of General Internal Medicine, Innsbruck Medical University, A-6020 Innsbruck, Austria*

**Abstract**

Antibodies directed to cyclic citrullinated peptides (anti-CCP) are highly specific for rheumatoid arthritis (RA) and can easily be detected in sera by using commercially available immunoassays. The second version of the anti-CCP test (anti-CCP2) demonstrated high specificity (89–98%) and good sensitivity (41–88%) for RA. Commercially available ELISA methods from three different companies are on the market. All three CCP2 assays show similar results as all CCP2 assays use the same antigen-coated plates. This study was an evaluation of a new automated method for the determination of anti-CCP2 in a routine laboratory setting. Five hundred and fourty three serum samples were tested for anti-CCP2 within normal routine diagnostic using a commercially available ELISA and retested with a prelaunch version of a new and fully-automated method (*EliA*™). The results were comparable. The new automated assay is easy to use and demonstrated a diagnostic sensitivity of 80% and specificity of 97%.

**Keywords:** *Anti-CCP, automated determination, rheumatoid arthritis, enzyme-linked immunosorbent assays*

**Abbreviations:** *ACR, American College of Rheumatology; AFA, antifilaggrin antibodies; AKA, antikeratin antibodies; anti-CCP, anti cyclic citrullinated peptide; APF, antiperinuclear factor; ELISA, enzyme-linked immunosorbent assays; RA, rheumatoid arthritis; RF, rheumatoid factor*

### Introduction

Rheumatoid arthritis (RA) is the most common inflammatory joint disease with a prevalence between 0.5 and 1% worldwide (Lawrence et al. 1998). The diagnosis is mainly based on clinical signs and symptoms according to latest recommendations in 1987 (Arnett et al. 1988). Within these seven diagnostic criterias, detection of IgM rheumatoid factor (RF) in serum is the only recommended laboratory marker. Though IgM-RF is measured in most studies and is the most often ordered autoantibody test in laboratory diagnosis, its specificity for diagnosing RA is limited. At very low levels IgM-RF is present in sera of most people. High concentrations of IgM-RF are not only detected in RA but also in other conditions with polyclonal stimuli to B-cells like viral and bacterial infections or chronic inflammations other than RA. The need for a better laboratory marker with a higher disease-related specificity and sensitivity was always evident.

A very specific antibody for RA was first described by Nienhuis and Mandema (1964) which is called antiperinuclear factor (APF) as these antibodies bound to constituents of the keratohyaline granules which are located close to the nucleus of buccal mucosa cells of adult people. The APF showed an acceptable sensitivity and compared to RF a much higher specificity (Hoet et al. 1991). Nevertheless, the test was never used for routine testing in case of practical inconvenience. The test required carefully selected buccal mucosa cells which were differentiated enough to contain the perinuclear factor. Experienced laboratory technicians were needed to perform the test based on indirect immunofluorescence and to recognize the different immunofluorescent patterns.

In 1979, another group of RA specific antibodies, the so-called antikeratin antibodies (AKA) was described (Young et al. 1979). The AKA bind to keratin-like structures in the cornified layer of stratum

Correspondence: M. Herold, Innsbruck Medical University, Clinical Department of Internal Medicine, Anichstrasse 35, A-6020 Innsbruck, Austria. Tel: 43 512 504 23321. Fax: 43 512 504 24213. E-mail: manfred.herold@uibk.ac.at

ISSN 1740-2522 print/ISSN 1740-2530 online © 2005 Taylor & Francis Group Ltd
DOI: 10.1080/14767050500134217

corneum. These autoantibodies could easily be detected by indirect immunofluorescence on rat oesophagus cryostat sections and some laboratories started to offer AKA measurement within normal routine diagnostics. The specificity for RA was comparable to APF (Hoet and van Venrooij 1992) and it could be demonstrated that AKA also precede the onset of RA (Kurki et al. 1992).

It was shown that APF and AKA target the same antigen (Sebbag et al. 1995) identified as the epithelial protein filaggrin (filament aggregating protein) which is involved in the organization of cytoskeletal structures. Several filaggrin subunits result from proteolytical cleavage of profilaggrin during differentiation of epithelial cells. Profilaggrin is first dephosphorylated and about 20% of the basic arginine residues are converted into neutral citrulline residues by the enzyme peptidylarginine deiminase. It has been documented that the modification to a citrulline containing protein is essential for the autoantigenicity of filaggrin (Schellekens et al. 1998) and citrullinated fillaggrin is the antigen targeted by APF and AKA. Immunoblotting assays and enzyme-linked immunosorbent assays (ELISA) using filaggrin purified from human skin as antigen detected antifilaggrin antibodies (AFA) in 42% of patients with RA with a specificity of 99% (Vincent et al. 1998). Using *in vitro* deiminated recombinant filaggrin increased the number of positive RA sera to 52% (Aho et al. 1999). Sensitivity and specificity of AFA seemed to be dependent on the method of filaggrin purification and on the difficulty to obtain antigen preparations with reproducable citrullin content. This technical problem could be solved by using isolated citrullinated filaggrin peptides as antigen but it became apparent that sera from different RA patients show different patterns of reactivity indicating the heterogenity of the autoimmune response. Using a combination of nine citrullinated peptide variants a sensitivity of 76% and a specificity of 96% were detected. The test was improved and simplified using a cyclic variant of a citrullinated peptide called anti-cyclic citrullinated peptide (CCP) test. This first anti-CCP test revealed a high diagnostic specificity of about 98% and a sensitivity around 70% (Boekel et al. 2002). To improve, the anti-CCP test peptides from dedicated libraries of citrullinated peptides were tested with RA sera to select the most reactive species. The selected peptides were made cyclic to ensure the exposure of the antigenic citrulline structure. This investigation resulted in the second generation anti-CCP assay which is sold worldwide as CCP2 assay (Vossenaar and Venrooij 2004).

Till the end of 2003, there were three distributors (Euro-Diagnostica, Arnhem, The Netherlands; Axis-Shield Diagnostics Ltd., Dundee, UK; Inova Diagnostics Inc., San Diego, CA) selling the CCP2 test. CCP2 is a brand name and all commercial available immunoassays use the same antigen and the same antigen-coated plates but differ in working procedures. The results of all three assays are similar (Dubucquoi et al. 2004, Garcia-Berrocal et al. 2005). The anti-CCP test has a comparable sensitivity but a much higher specificity than the IgM-RF test in diagnosing RA (Table I) and it is not surprising that anti-CCP testing has increasing acceptance in laboratory medicine. In November 2004, Sweden Diagnostics affiliate of Pharmacia Diagnostics AB Freiburg, Germany, launched the first fully-automated testing system for CCP antibodies called *ELIA*™ CCP. This assay was tested in a prelaunch version on samples collected consecutively from patients coming to the Rheumatology Unit of our hospital.

### Materials and methods

Five hundred and forty three serum samples (395 female, 148 male) from outpatients of our hospital were tested for anti-CCP routinely by a commercially available ELISA (Inova Diagnostics Inc.) and retested

Table I. Sensitivity and specificity of anti-CCP and IgM-RF antibody measurements in sera of patients with RA.

| | Anti-CCP | | IgM-Rf | |
|---|---|---|---|---|
| Source | Sensitivity (%) | Specificity (%) | Sensitivity (%) | Specificity (%) |
| Schellekens et al. (2000) | 68 | 98 | 54 | 91 |
| Goldbach-M. et al. (2000) | 50 | 90 | 66 | 87 |
| Bizzaro et al. (2001) | 41 | 98 | 62 | 84 |
| Bas et al. (2002) | 68 | 96 | 75 | 74 |
| Jansen et al. (2002) | 43 | 98 | 50 | 93 |
| Lee and Schur (2003) | 66 | 90 | 72 | 80 |
| Suzuki et al. (2003) | 88 | 89 | 70 | 82 |
| Zeng et al. (2003) | 47 | 97 | 59 | |
| Saraux et al. (2003) | 47 | 93 | 41 | |
| Araki et al. (2004) | 81 | 92 | | |
| Dubucquoi et al. (2004) | 65 | 96 | 60 | 70 |
| Girelli et al. (2004) | 71 | 95 | 91 | 31 |
| Vallbracht et al. (2004) | 64 | 97 | 66 | 82 |

bulkwise by *EliA*™-CCP (Sweden Diagnostics affiliate of Pharmacia Diagnostics AB Freiburg), an automated test system for the determination of anti-CCP. The tests of both companies correspond to the so-called anti-CCP2 test. Results were classified according to the recommendations of the manufacturers. The ELISA from Inova categorizes negative (<20 U/ml), weak positive (20–39 U/ml), moderate positive (40–59 U/ml) and strong positive (>60 U/ml). *EliA*™ from Pharmacia gave a preliminary recommendation with negative (<7 U/ml), borderline positive (7–10 U/ml) and positive (>10 U/ml).

Serum samples were drawn from patients consecutively coming to our hospital with joint problems. CCP was routinely tested in all patients with clinical suspect for RA or other inflammatory joint diseases and from patients with prediagnosed RA but not yet tested on anti-CCP. After detection of anti-CCP with both methods, patients' files were screened to evaluate the diagnosis. RA was diagnosed according to the revised ACR criteria (Arnett et al. 1998).

### Results

Overall, there was a good agreement in the results of both assays (Table II). Among the positive samples detected by ELISA, 12 samples were negative by *EliA*™. These samples were from patients who were diagnosed in 1 case as possible RA, in 4 cases as non-RA and in 7 cases a diagnose was not known.

Thirteen out of 88 positive samples tested by *EliA*™ were negative in the Inova assay. Twelve of these samples were only weak positive with concentrations below 29 U/ml and only one sample from a patient diagnosed as non-RA was strong positive. The 12 weak positive samples were in 8 cases from patients with RA (Table III). Three samples were moderate to strong positive in ELISA but negative in *EliA*™. None of these three samples was from a patient with definite or possible RA.

In sera of 66 patients with definite RA (Table IV), the ELISA and *EliA*™ revealed 45 (68%) and 53 (80%) positive results, respectively. In the group of patients with possible RA, anti-CCP results were identical. In the non-RA group results were almost identical with 11 positive and 317 negative samples in ELISA versus 10 positive and 318 negative samples in *EliA*™. The diagnostic sensitivity and specificity were 68.2 and 96.9% in the ELISA and 80.3 and 97.0% in the *EliA*™, respectively.

Sera were collected from 16 patients at two different times and analysed blinded. All results were highly reproducible in both assay systems though all samples were analyzed in different assay runs.

Table II. Number of serum samples with negative, borderline or weak positive and positive results comparing ELISA and *EliA*™.

| | Automated (*EliA*™ Pharmacia) | | |
|---|---|---|---|
| ELISA Inova | Negative | Borderline | Positive |
| Negative | 442 | 1 | 13 |
| Weak positive | 9 | 0 | 7 |
| Moderate positive | 2 | 0 | 5 |
| Strong positive | 1 | 0 | 63 |

The ELISA from Inova categorizes negative <20 U/ml, weak positive 20–39 U, moderate positive 40–59 U and strong positive (>60 U). The automated *EliA*™ from Sweden Diagnostics categorizes negative <7 U, borderline 7–10 U and positive >10 U.

Table III. Incidence of definite RA in those patients whose sera revealed different results in both anti-CCP2 assays.

| | | | Rheumatoid arthritis | | | |
|---|---|---|---|---|---|---|
| ELISA | *EliA*™ | *n* | Yes | Possible | No | Unknown |
| Negative | Positive | 13 | 8 | 1 | 2 | 2 |
| Positive | Negative | 3 | – | – | 1 | 2 |

### Discussion

Antibodies directed to citrullinated proteins and their association to RA are well known since several years. These antibodies occur in patients with RA and can even be detected in sera years before onset of disease symptoms (Kurki et al. 1992, Rantapaa-Dahlqvist et al. 2003, Nielen et al. 2004). The development of synthetic citrullinated proteins provided the use as antigens in immunoassays. Since test improvement by making cyclic variants of citrullinated peptides as antigens in commercially available ELISAs, there is an increasing interest in testing these antibodies as diagnostic and prognostic markers in patients with RA (Kroot et al. 2000, Schellekens et al. 2000, Vasishta 2002, Bas et al. 2003, Meyer et al. 2003, Vencovsky et al. 2003, Berglin et al. 2004, Forslind et al. 2004, van Gaalen et al. 2004a,b, Kastbom et al. 2004, Lopez-Hoyos 2004, Maddali Bongi et al. 2004, van Venrooij et al. 2004, Lindqvist et al. 2005, Nielen et al. 2005, Raza et al. 2005).

In 2004, there were three commercial second-generation ELISAs for the measurement of anti-CCP antibodies marketed as anti-CCP2 tests. All CCP-2

Table IV. Incidence of positive or negative results of anti-CCP antibodies in serum samples of different patient groups.

| | | ELISA anti-CCP | | *EliA*™ anti-CCP | |
|---|---|---|---|---|---|
| Diagnosis | *n* | Positive | Negative | Positive | Negative |
| RA | 66 | 45 | 21 | 53 | 13 |
| Possible RA | 42 | 12 | 30 | 12 | 30 |
| Non RA | 328 | 11 | 317 | 10 | 318 |
| Unknown | 107 | 19 | 88 | 14 | 93 |
| Total | 543 | 87 | 456 | 89 | 454 |

tests use the same second-generation synthetic citrullinated peptide antigen bound to the same surface of a microwell plate. The tests differ in handling procedures and other components of the assay system but reveal similar results. Comparing to one of these three ELISA's, we tested a new and fully automated CCP-2 assay in a prelaunch version. The results were comparable and both assays showed the same specificity. We found some difference between the two assays in sensitivity. This difference is mainly based on 8 samples of RA patients, which were negative in the ELISA and weak positive in the *EliA*™. Comparing to data from literature, the sensitivity of 80% for the automated assay fits to results from anti-CCP2 ELISAs with optimum cut-off values (Garcia-Berrocal et al. 2005).

IgM rheumatoid factor has been commonly used as serological marker of RA. In all publications, anti-CCP demonstrated a higher specificity and a comparable or slightly higher sensitivity than IgM-RF. There is no doubt that anti-CCP is a valuable serum marker for the diagnosis of RA. Different analytical procedures may be one of the reasons that measurement of IgM-RF is still offered in most laboratories but not anti-CCP. IgM-RF is usually measured automatically on common analyzers, which are used in routine laboratory diagnostics. *EliA*™ anti-CCP is the first fully automated method for the determination of anti-CCP with all advantages of an automated analytical method like standardized conditions and short hands-on-time.

The new automated immunoassay *EliA*™ for measuring anti-CCP is easy to use and shows reliable results with sensitivity and specificity for diagnosing RA comparable to given anti-CCP2 ELISAs on the market.

### References

Aho K, Palosuo T, Lukka M, Kurki P, Isomaki H, Kautiainen H, von Essen R. 1999. Anti-filaggrin antibodies in recent-onset arthritis. Scand J Rheumatol 28:113–116.

Araki C, Hayashi N, Moriyama M, Morinobu S, Mukai M, Koshiba M, Kawano S, Kumagai S. 2004. Usefulness of anti-cyclic citrullinated peptide antibodies (anti-CCP) for the diagnosis of rheumatoid arthritis. Rinsho Byori 52:966–972.

Arnett FC, Edworthy SM, Bloch DA, McShane DJ, Fries JF, Cooper NS, Healey LA, Kaplan SR, Liang MH, Luthra HS, et al. 1988. The American Rheumatism Association 1987 revised Criteria for the classification of rheumatoid arthritis. Arthritis Rheum 31:315–324.

Bas S, Perneger TV, Seitz M, Tiercy JM, Roux-Lombard P, Guerne PA. 2002. Diagnostic tests for rheumatoid arthritis: Comparison of anti-cyclic citrullinated peptide antibodies, anti-keratin antibodies and IgM rheumatoid factors. Rheumatology 41:809–814.

Bas S, Genevay S, Meyer O, Gabay C. 2003. Anti-cyclic citrullinated peptide antibodies, IgM and IgA rheumatoid factors in the diagnosis and prognosis of rheumatoid arthritis. Rheumatology 42:677–680.

Berglin E, Padyukov L, Sundin U, Hallmans G, Stenlund H, Van Venrooij WJ, Klareskog L, Dahlqvist SR. 2004. A combination of autoantibodies to cyclic citrullinated peptide (CCP) and HLA-DRB1 locus antigens is strongly associated with future onset of rheumatoid arthritis. Arthritis Res Ther 6:R303–R308, Epub 2004 May 11.

Bizzaro N, Mazzanti G, Tonutti E, Villalta D, Tozzoli R. 2001. Diagnostic accuracy of the anti-citrulline antibody assay for rheumatoid arthritis. Clin Chem 47:1089–1093.

Boekel MAM, Vossenaar ER, van den Hoogen FHJ, van Venrooij WJ. 2002. Autoantibody systems in rheumatoid arthritis: Specificity, sensitivity and diagnostic value. Arthritis Res 4:87–93.

Dubucquoi S, Solau-Gervais E, Lefranc D, Marguerie L, Sibilia J, Goetz J, Dutoit V, Fauchais AL, Hachulla E, Flipo RM, Prin L. 2004. Evaluation of anti-citrullinated filaggrin antibodies as hallmarks for the diagnosis of rheumatic diseases. Ann Rheum Dis 63:415–419.

Forslind K, Ahlmen M, Eberhardt K, Hafstrom I, Svensson B, BARFOT Study Group. 2004. Prediction of radiological outcome in early rheumatoid arthritis in clinical practice: Role of antibodies to citrullinated peptides (anti-CCP). Ann Rheum Dis 63:1090–1095.

van Gaalen FA, van Aken J, Huizinga TW, Schreuder GM, Breedveld FC, Zanelli E, van Venrooij WJ, Verweij CL, Toes RE, de Vries RR. 2004a. Association between HLA class II genes and autoantibodies to cyclic citrullinated peptides (CCPs) influences the severity of rheumatoid arthritis. Arthritis Rheum 50:2113–2121.

van Gaalen FA, Linn-Rasker SP, van Venrooij WJ, de Jong BA, Breedveld FC, Verweij CL, Toes RE, Huizinga TW. 2004b. Autoantibodies to cyclic citrullinated peptides (CCP) predict progression to rheumatoid arthritis in patients with undifferentiated arthritis. Arthritis Rheum 50:709–715.

Garcia-Berrocal B, Gonzalez C, Perez M, Navajo JA, Moreta I, Davila C, Gonzalez-Buitrago JM. 2005. Anti-cyclic citrullinated peptide autoantibodies in IgM rheumatoid factor-positive patients. Clin Chim Acta 354:123–130.

Girelli F, Foschi FG, Bedeschi E, Calderoni V, Stefanini GF, Martinelli MG. 2004. Is anti cyclic citrullinated peptide a useful laboratory test for the diagnosis of rheumatoid arthritis? Allerg Immunol 36:127–130.

Goldbach-Mansky R, Lee J, McCoy A, Hoxworth J, Yarboro C, Smolen JS, Steiner G, Rosen A, Zhang C, Menard HA, Zhou ZJ, Palosuo T, Van Venrooij WJ, Wilder RL, Klippel JH, Schumacher Jr, HR, El-Gabalawy HS. 2000. Rheumatoid arthritis associated autoantibodies in patients with synovitis of recent onset. Arthritis Res 2:236–243.

Hoet RM, van Venrooij WJ. 1992. The perinuclear factor and antikeratin antibodies in rheumatoid arthritis. In: Smolen J, Kalden J, Maini RN, editors. Rheumatoid arthritis. Berlin: Springer Verlag. pp 299–318.

Hoet RM, Boerbooms AM, Arends M, et al. 1991. Antiperinuclear, a marker autoantibody for rheumatoid arthritis: Colocalisation of the perinuclear factor and profilagrin. Ann Rheum Dis 50:611–618.

Jansen AL, van der Horst-Bruinsma I, van Schaardenburg D, van de Stadt RJ, de Koning MH, Dijkmans BA. 2002. Rheumatoid factor and antibodies to cyclic citrullinated peptide differentiate rheumatoid arthritis from undifferentiated polyarthritis in patients with early arthritis. J Rheumatol 29:2034–2040.

Kastbom A, Strandberg G, Lindroos A, Skogh T. 2004. Anti-CCP antibody test predicts the disease course during 3 years in early rheumatoid arthritis (the Swedish TIRA project). Ann Rheum Dis 63:1085–1089.

Kroot EJ, de Jong BA, van Leeuwen MA, Swinkels H, van den Hoogen FH, van't Hof M, van de Putte LB, van Rijswijk MH, van Venrooij WJ, van Riel PL. 2000. The prognostic value of anti-cyclic citrullinated peptide antibody in patients with recent-onset rheumatoid arthritis. Arthritis Rheum 43:1831–1835.

Kurki P, Aho K, Palosuo T, Heliövaara M. 1992. Immunopathology of rheumatoid arthritis. Antikeratin antibodies precede the clinical disease. Arthritis Rheum 35:914–917.

Lawrence RC, Helmick CG, Arnett FC, et al. 1998. Estimates of the prevalence of arthritis and selected musculoskeletal disorders in the United States. Arthritis Rheum 41:778–799.

Lee DM, Schur PH. 2003. Clinical utility of the anti-CCP assay in patients with rheumatic diseases. Ann Rheum Dis 62:870–874.

Lindqvist E, Eberhardt K, Bendtzen K, Heinegard D, Saxne T. 2005. Prognostic laboratory markers of joint damage in rheumatoid arthritis. Ann Rheum Dis 64:196–201.

Lopez-Hoyos M, Ruiz de Alegria C, Blanco R, Crespo J, Pena M, Rodriguez-Valverde V, Martinez-Taboada VM. 2004. Clinical utility of anti-CCP antibodies in the differential diagnosis of elderly-onset rheumatoid arthritis and polymyalgia rheumatica. Rheumatology 43:655–657.

Maddali Bongi S, Manetti R, Melchiorre D, Turchini S, Boccaccini P, Vanni L, Maggi E. 2004. Anti-cyclic citrullinated peptide antibodies are highly associated with severe bone lesions in rheumatoid arthritis anti-CCP and bone damage in RA. Autoimmunity 37:495–501.

Meyer O, Labarre C, Dougados M, Goupille P, Cantagrel A, Dubois A, Nicaise-Roland P, Sibilia J, Combe B. 2003. Anticitrullinated protein/peptide antibody assays in early rheumatoid arthritis for predicting five year radiographic damage. Ann Rheum Dis 62:120–126.

Nielen MM, van Schaardenburg D, Reesink HW, van de Stadt RJ, van der Horst-Bruinsma IE, de Koning MH, Habibuw MR, Vandenbroucke JP, Dijkmans BA. 2004. Specific autoantibodies precede the symptoms of rheumatoid arthritis: A study of serial measurements in blood donors. Arthritis Rheum 50:380–386.

Nielen MM, van der Horst AR, van Schaardenburg D, van der Horst-Bruinsma IE, van de Stadt RJ, Aarden L, Dijkmans BA, Hamann D. 2005. Antibodies to citrullinated human fibrinogen (ACF) have diagnostic and prognostic value in early arthritis. Ann Rheum Dis Jan 7 [Epub ahead of print].

Nienhuis RLF, Mandema EA. 1964. A new serum factor in patients with rheumatoid arthritis. The perinuclear factor. Ann Rheum Dis 23:302–305.

Rantapaa-Dahlqvist S, de Jong BA, Berglin E, Hallmans G, Wadell G, Stenlund H, Sundin U, van Venrooij WJ. 2003. Antibodies against cyclic citrullinated peptide and IgA rheumatoid factor predict the development of rheumatoid arthritis. Arthritis Rheum 48:2741–2749.

Raza K, Breese M, Nightingale P, Kumar K, Potter T, Carruthers DM, Situnayake D, Gordon C, Buckley CD, Salmon M, Kitas GD. 2005. Predictive value of antibodies to cyclic citrullinated peptide in patients with very early inflammatory arthritis. J Rheumatol 32:231–238.

Saraux A, Berthelot JM, Devauchelle V, Bendaoud B, Chales G, Le Henaff C, Thorel JB, Hoang S, Jousse S, Baron D, Le Goff P, Youinou P. 2003. Value of antibodies to citrulline-containing peptides for diagnosing early rheumatoid arthritis. J Rheumatol 30:2535–2539.

Schellekens GA, de Jong BAW, van den Hoogen FHJ, van de Putte LBA. 1998. Citrulline is an essential constituent of antigenic determinants recognized by rheumatoid arthritis-specific autoantibodies. J Clin Investig 101:273–281.

Schellekens GA, Visser H, de Jong BA, van den Hoogen FH, Hazes JM, Breedveld FC, van Venrooij WJ. 2000. The diagnostic properties of rheumatoid arthritis antibodies recognizing a cyclic citrullinated peptide. Arthritis Rheum 43:155–163.

Sebbag M, Simon M, Vincent C, Masson-Bessiere C, Girbal E, Durieux JJ, Serre G. 1995. The antiperinuclear factor and the so-called antikeratin antibodies are the same rheumatoid arthritis-specific autoantibodies. J Clin Investig 95:2672–2679.

Suzuki K, Sawada T, Murakami A, Matsui T, Tohma S, Nakazono K, Takemura M, Takasaki Y, Mimori T, Yamamoto K. 2003. High diagnostic performance of ELISA detection of antibodies to citrullinated antigens in rheumatoid arthritis. Scand J Rheumatol 32:197–204.

Vallbracht I, Rieber J, Oppermann M, Forger F, Siebert U, Helmke K. 2004. Diagnostic and clinical value of anti-cyclic citrullinated peptide antibodies compared with rheumatoid factor isotypes in rheumatoid arthritis. Ann Rheum Dis 63:1079–1084.

Vasishta A. 2002. Diagnosing early-onset rheumatoid arthritis: The role of anti-CCP antibodies. Am Clin Lab 21:34–36.

Vencovsky J, Machacek S, Sedova L, Kafkova J, Gatterova J, Pesakova V, Ruzickova S. 2003. Autoantibodies can be prognostic markers of an erosive disease in early rheumatoid arthritis. Ann Rheum Dis 62:427–430.

van Venrooij WJ, Vossenaar ER, Zendman AJ. 2004. Anti-CCP antibodies: The new rheumatoid factor in the serology of rheumatoid arthritis. Autoimmun Rev 3(Suppl 1):S17–S19.

Vincent C, Simon M, Sebbag M, Girbal-Neuhauser E, Durieux JJ, Cantagrel A, Fournie B, Mazieres B, Serre G. 1998. Immunoblotting detection of autoantibodies to human epidermis filaggrin: A new diagnostic test for rheumatoid arthritis. J Rheumatol 25:838–846.

Vossenaar ER, Venrooij WJ. 2004. Anti-CCP antibodies, a specific marker for (early) rheumatoid arthritis. Clin Appl Immunol Rev 4:239–262.

Young BVJJ, Mallaya RK, Leslie RDG, Clark CJM, Hamblin TJ. 1979. Antikeratin antibodies in rheumatoid arthritis. Br Med J 2:97–99.

Zeng X, Ai M, Tian X, Gan X, Shi Y, Song Q, Tang F. 2003. Diagnostic value of anti-cyclic citrullinated peptide antibody in patients with rheumatoid arthritis. J Rheumatol 30:1451–1455.

